# Electrophysiological characteristics of epicardial breakthrough during catheter ablation of perimitral atrial flutter

**DOI:** 10.3389/fcvm.2022.1030916

**Published:** 2022-11-17

**Authors:** Chheng Chhay, Chu-Yu Hsu, Shih-Lin Chang, Yenn-Jiang Lin, Li-Wei Lo, Yu-Feng Hu, Fa-Po Chung, Ting-Yung Chang, Chin-Yu Lin, Yuan Hung, Chih-Min Liu, Ling Kuo, Shin-Huei Liu, Lia Ahli, Ming-Jen Kuo, Wen-Han Cheng, Pei-Heng Kao, Wei-Tso Chen, Thien Chuong-Nguyen Khac, Wei-Shiang Lin, Shih-Ann Chen

**Affiliations:** ^1^Heart Rhythm Center and Division of Cardiology, Department of Medicine, Taipei Veterans General Hospital, Taipei, Taiwan; ^2^Cardiovascular Department, Faculty of Medicine, University of Health Sciences, Phnom Penh, Cambodia; ^3^Division of Cardiology, Department of Internal Medicine, Taoyuan Armed Forces General Hospital, Taoyuan, Taiwan; ^4^Division of Cardiology, Department of Internal Medicine, Tri-Service General Hospital, National Defense Medical Center, Taipei, Taiwan; ^5^Faculty of Medicine, School of Medicine, National Yang Ming Chiao Tung University, Taipei, Taiwan; ^6^Cardiovascular Research Center, National Yang Ming Chiao Tung University, Taipei, Taiwan; ^7^Taichung Veterans General Hospital, Taichung, Taiwan

**Keywords:** perimitral atrial flutter, EGM interval of distal CS, epicardial breakthrough, catheter ablation, atrial fibrillation

## Abstract

**Introduction:**

Unsuccessful endocardial ablation for perimitral atrial flutter (AFL) could be attributed by the epicardial bridging.

**Objective:**

This study aimed to investigate the electrophysiological characteristics of epicardial breakthrough during catheter ablation of perimitral AFL.

**Materials and methods:**

This retrospective study recruited 40 patients who received successful catheter ablation of perimitral AFL from January 2016 to June 2021. The patients were divided into two groups: group 1 (*n* = 18) successful endocardial ablation, and group 2 (*n* = 22) successful epicardial ablation following unsuccessful endocardial ablation owing to incomplete mitral block or unachievable termination AFL. The local electrogram (EGM) interval of coronary sinus (CS) duration perimitral AFL was measured before catheter ablation.

**Results:**

There was no significant difference in the baseline characteristics between the two groups. In group 2, 60% of successful epicardial ablation was performed in intra-CS ablation and 40% in VOM ethanol infusion. Group 2 patients had a longer EGM interval of distal CS than that in group 1 (CS1-2: 64.2 17.5 vs. 42.4 0.09 ms, *P* = 0.008, CS3-4: 57.13 19.4 vs. 43.8 7.5 ms; *P* = 0.001). The conduction velocity at successful site was slower in group 2 compared to group 1 (0.18 0.05 vs. 0.75 0.19 m/s, *P* = 0.040). In the multivariate analysis, distal EGM interval (CS1-2) was identified as independent predictor of the need of epicardial ablation with the optimal cutoff of 49 ms.

**Conclusion:**

Longer EGM interval in distal CS during perimitral AFL was observed in perimitral AFL patients with epicardial breakthrough following endocardial-failed ablation, which may be associated with the need of epicardial ablation.

## Introduction

Macroreentrant perimitral atrial flutter (AFL) could occur in patients with atrial fibrillation (AF) who received catheter ablation of AF with circumferential pulmonary vein isolation (PVI) (1, 2). The conventional ablation technique of perimitral AFL was to create a posterior mitral line consisting of point-by-point ablation from the lateral mitral annulus to the ostium of the left inferior pulmonary veins (PV) (3). Unsuccessful endocardial ablation for perimitral AFL could be caused by the epicardial bridging which across structures and regions involving Bachmann’s region, septopulmonary bundle, coronary sinus, and vein of Marshall (VoM). Coronary sinus to left atrial connections were observed in more than one-third of cases of left atrial (LA) AFL involving epicardial anatomic regions (4). High-density mapping facilitates higher resolution to delineate site of epicardial breakthrough involving in a complex circuit. Confirmation of discontinuous activation patterns that indicate 3-D propagation with attention to corresponding regional LA anatomy may decrease the incidence of ablation failures for complex reentry (4).

However, the electrophysiological properties between patients with endocardial and epicardial successful ablation of perimitral AFL are not investigated. The purpose of this study was to investigate the electrophysiological characteristics of epicardial breakthrough during catheter ablation of perimitral AFL.

## Materials and methods

### Study population

**FIGURE 1:**
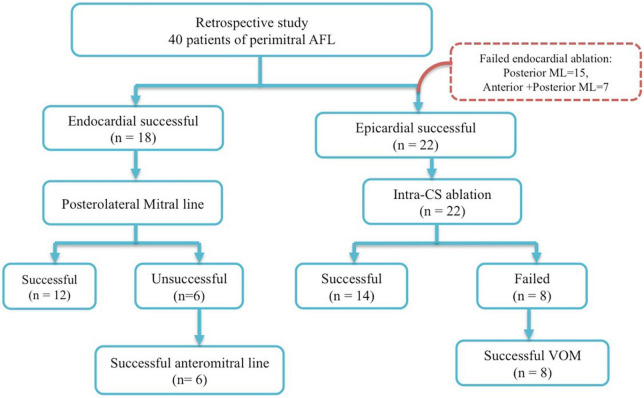
Study design.

This study retrospectively recruited 40 consecutive patients undergoing radiofrequency catheter ablation of perimitral AFL from the Heart Rhythm Center of Taipei Veterans General Hospital, during January 2016 to June 2021. The study patients were divided into two groups: group 1 (*n* = 18) consisted of patients with successful endocardial ablation, and group 2 (*n* = 22) contained patients with successful epicardial ablation following unsuccessful endocardial ablation. In group 1, 12 patients of perimitral AFL have achieved successful ablation at posteromitral line and six patients failed. Six patients had successful mitral line block after anteromitral line following unsuccessful ablation at posteromitral line. In group 2, 22 patients had unsuccessful ablation at endocardial sites. Fourteen patients had mitral bidirectional block after ablation of intra-CS distal. Eight patients had successful mitral bidirectional block after ethanol infusion of VoM following failure intra-CS ablation. Patients with unsuccessful ablation of perimitral AFL (no bidirectional mitral isthmus block) were excluded from this study (Figure 1). The study protocol was approved by the Institutional Review Board at Taipei Veterans General Hospital, Taipei, Taiwan.

### Electrophysiological study and mapping strategy of atrial fibrillation

In our study, 65% of patients had received prior AF ablation. The method and procedure of AF ablation have been described in our previous studies (5–7). In brief, electro-anatomic mappings were guided and constructed by the Advisor™ HD grid/Ensite Navigation System (St. Jude Medical, Minneapolis, MN, USA), PENTARAY^®^/CARTO^®^ mapping system (Biosense-Webster Inc.; Diamond Bar, CA, USA), or Orion™/Rhythmia™ (Boston Scientific Corporation, Middlesex Country, MA, USA). All antiarrhythmic agents, except amiodarone, were stopped for more than five half-lives before the procedure. Patients received either circumferential or segmental PVI *via* an open (Cool Path, St. Jude Medical) irrigated tip ablation catheter, with radiofrequency energy set to 25–35 W and 40 s for each lesion, with temperature 35–40°C. Additional linear ablations, or complex fractionated atrial electrogram (CFAE) ablation, were performed conditionally, at the operators’ discretion. AF was tried to be identified after infusion of isoproterenol (1–4 μg/min), intermittent burst pacing from the right atrium (RA) and coronary sinus (CS), or after an intravenous bolus of adenosine.

### Diagnosis of perimitral atrial flutter and ablation strategy

Electrophysiology study and mapping have been described in our previous study (8–12). In brief, all patients stopped taking antiarrhythmic agents (except amiodarone) for >5 half-lives before the procedure, and each of them underwent electrophysiological study and catheter ablation under fasting and non-sedative states. After atrial trans-septal puncture, electro-anatomic maps were constructed using three-dimensional mapping system in all patients by the Advisor™ HD grid/Ensite Navigation System (St. Jude Medical, Minneapolis, MN, USA), PENTARAY^®^/CARTO^®^ mapping system (Biosense-Webster Inc.; Diamond Bar, CA, USA), or Orion™/Rhythmia™ (Boston Scientific Corporation, Middlesex Country, MA, USA), and irrigated ablation catheter was used at the discretion of treating electrophysiology.

Perimitral AFL was diagnosed with a three-dimensional anatomic mapping system with the entrainment pacing technique. Bipolar voltage was obtained at the mitral isthmus (lateral: ostium of LIPV to lateral mitral annulus; medial: ostium of RIPV to medial mitral annulus) (13). The difference between the post-pacing interval (PPI) and tachycardia cycle length (TCL) <20 ms from posteromitral line at 4 and 12 o’clock sites in the LA or CS along mitral annulus was considered as a perimitral AFL. The epicardial-endocardial breakthrough (EEB) was defined as (i) the presence of a focal endocardial activation with a radial spreading, (ii) present with the same timing on every tachycardia cycle length (14), and (iii) discontinuous wave front of endocardial activation with a gap in activation resulting in focal endocardial breakout distally and activation back toward the gap (4). Bipolar and unipolar electrogram at the site of breakthrough site were analyzed to confirm the wavefront activation. The fractionated bipolar electrogram at the endocardial focal breakthrough site with unipolar electrograms at this site was also analyzed.

Subsequently, radiofrequency catheter ablation (RFCA) was applied from the posterolateral mitral line at 4 o’clock position of the MA to the bottom LIPV using 4-mm open-irrigated catheter using a power control mode with a maximal power of 30–40 W. If the standard posterior mitral line could not achieve, then anteromitral line ablation was performed at the discretion of the ablator. If the endocardial ablation was unable to successfully reach a complete MI conduction block (defined by bidirectional block across the MI line) or terminated AFL, further RFCA application (20–25 W with irrigated ablation catheter) was delivered within the CS opposite of the endocardial MI line. VoM ethanol injection was performed if intra-CS ablation was not achieved. The endpoint of ablation was defined as a complete block of posterolateral mitral line or anteromitral line (proved by bidirectional conduction block across the perimitral ablation line). “Endocardial successful ablation” was defined as the elimination of perimitral AFL with bidirectional block of perimitral ablation line. “Epicardial successful ablation” was defined as the elimination of perimitral AFL with bidirectional block of posterior mitral isthmus by means of intra-CS ablation or VoM ethanol infusion.

### Measurement of local electrogram intervals in coronary sinus

Prior studies had demonstrated the involvement of coronary sinus to LA connections in atrial tachycardia and AFL. Endocardial ablation at the LSPV-LAA ridge was successful in ablating of MAFs associated with EB involving the VoM (Tung’s group: >80%; Vlachos group: >73%), and the elimination of distal CS to LA connections also reduced atrial arrhythmia recurrences (4, 15).

The coronary sinus was cannulated in all cases using a decapolar coronary sinus catheter (Abbott, St. Paul, MN, USA) with 2-mm interelectrode distance and 5-mm space between two electrode pairs. The proximal pair of electrodes was positioned at the CS ostium, and the distal pair of electrodes was located at the lateral aspect of the great cardiac vein. Local EGM intervals of CS were measured from initial activation to electrogram termination during perimitral atrial flutter, and the percentage of CS local EGM was calculated by CS interval dividing tachycardia cycle length in both groups before ablation of mitral AFL, or VoM alcoholization (16). Local conduction velocity in CS was defined by the distance between the two adjacent points divided by the activation time difference according to the isochronal line that was aligned with the directional vectors by the coherent map, Carto mapping system (Biosense-Webster Inc.; Diamond Bar, CA, USA), or obtained with UHD mapping directly by the Ensite Navigation System (St. Jude Medical, Minneapolis, MN, USA) (17).

### Statistical analyses

Continuous variables were expressed as mean ± standard deviation and categorical variables as counts (percentages). The two-sample independent *t*-test was used to analyze continuous variables as appropriate. Categorical data were compared using a chi-square test with Yates correction or the Fisher exact test. Binary logistic regression was used for identifying the independent predictor of endocardial and epicardial successful ablation. A *P*-value of less than 0.05 was considered significant. Analysis was performed using IBM SPSS Statistics 22 (SPSS Inc., Chicago, IL, USA). To determine the optimal cutoff value of CS duration for differential diagnosis of epicardial and endocardial connection, receiver operating characteristic (ROC) curve analysis was performed. The optimal cutoff value was determined as the cutoff value with maximal percent efficiency.

## Results

### Patient characteristics

Patient characteristics are listed in Table 1. Eighteen patients had successful endocardial ablation (Group I), and twenty-two patients had successful epicardial ablation (Group 2) by intra-CS ablation or vein of Marshall ethanol infusion. Median age was not significantly different between the two group (56.1 12.2 vs. 55.8 10.1 year old, *P* = 0.93, respectively). Most of the patients in perimitral AFL were male (66.7% in group 1 and 90.9% in group 2, *P* = 0.06). There was no significant difference between two groups in the other baseline characteristics including BMI, hypertension, dyslipidemia, diabetes mellitus, smoking, thyroid disease, coronary artery disease, and renal failure. More than 45% of patients in both groups used more than two kinds of antiarrhythmic drug before receiving catheter ablation of mitral AFL.

**TABLE 1:** Baseline characteristics.

Baseline characteristics	Endocardial successful group (*N* = 18)	Epicardial successful group (*N* = 22)	*P*-value
Age	56.1 ± 12.2	55.81 ± 10.1	0.93
Gender (Male)	12 (66.7%)	20 (90.9%)	0.06
BMI	25.6 ± 3.4	25.0 ± 3.1	0.62
Hypertension	8 (44.4%)	9 (40.9%)	0.82
Dyslipidemia	2 (11.1%)	3 (13.6%)	0.81
Diabetes mellitus	4 (22.2%)	2 (9.1%)	0.25
Smoking	4 (22.2%)	4 (18.2%)	0.75
Thyroid disease	5 (27.8%)	2 (9.1%)	0.12
Coronary artery disease	4 (22.2%)	2 (9.1%)	0.25
Renal failure	2 (11.1%)	0 (%)	0.11
Anti-arrhythmic agent ≥ 2	9 (50%)	10(45.4%)	0.28
Prior AF ablation	13 (72.2%)	13 (59.1%)	0.39
**Pre-ablation echocardiography parameters**			
LA diameter (mm)	41.5 ± 9.11	42.3 ± 7.2	0.76
LVEF (%)	59.6 ± 6.1	58.2 ± 4.6	0.45
LVIDd (mm)	46.8 ± 6.3	48.6 ± 6.0	0.40
LVIDs (mm)	29.3 ± 4.9	30.8 ± 7.3	0.49
MV E velocity (cm/s)	94.7 ± 26.6	82.9 ± 33.8	0.27
MV A velocity (cm/s)	61.8 ± 24.8	47.6 ± 15.7	0.08
E/A ratio	1.7 ± 0.9	1.7 ± 0.7	0.89
E’-Med (cm/s)	6.9 ± 2.6	8.1 ± 2.6	0.19
E/E’-med	15.8 ± 7.8	11.9 ± 9.8	0.21
**CS activation during mitral flutter**			
CS1-2 (ms)	42.4 ± 10.9	64.2 ± 17.5	**0.008**
CS1-2/TCL (%)	18.6 ± 6.0	25.3 ± 6.3	**0.001**
CS3-4 (ms)	43.8 ± 7.5	57.13 ± 19.4	**0.001**
CS3-4/TCL (%)	19.2 ± 5.4	23.7 ± 6.8	**0.03**
CS1-2∼CS3-4 (ms)	50.4 ± 8.5	69.8 ± 22.9	**0.001**
CS1-2∼CS3-4/TCL (%)	22.2 ± 6.3	29.0 ± 7.6	**0.004**
CS5-6 (ms)	40.5 ± 7.1	41.0 ± 12.8	0.89
CS5-6/TCL (%)	18.1 ± 5.5	17.64 ± 5.6	0.77
CS7-8 (ms)	43.2 ± 12.2	44.6 ± 16.1	0.76
CS7-8/TCL (%)	19.1 ± 6.3	19.3 ± 7.2	0.92
CS9-10 (ms)	40.4 ± 16.6	43.5 ± 20.1	0.61
CS9-10/TCL (%)	17.6 ± 7.1	18.3 ± 7.9	0.78
CS1_2 to 9_10	82.3 ± 15.6	97.6 ±	**0.01**
CS1_2 to 9_10/TCL (%)	33.9 ± 12.4	41.7 ± 7.6	**0.02**
**Procedure characteristics**			
Cycle length	234.0 ± 41.2	237.7 ± 27.0	0.74
Clockwise perimitral AFL	15 (83.3%)	18 (81.8%)	0.90
Complete circumferential 4PVs isolation	16 (88.9%)	17 (77.3%)	0.34
Posterior Mitral line	15 (83.3%)	19 (95.5%)	0.20
Anterior Mitral Line	4 (22.2%)	7 (31.8%)	0.50
Posterior + Anterior Mitral line	5 (27.8%)	6 (27.3%)	0.97
Roof line	6 (33.3%)	6 (27.3%)	0.68
Cavotricuspid isthmus	14 (77.8%)	16 (72.7%)	0.71
VOM alcohol	0 (0%)	8 (40.0%)	**0.004**
Intra-CS ablation	0 (0%)	14 (60.0%)	**0.00**
SVC isolation	2 (11.8%)	1 (4.5%)	0.40
LA volume (ml)	164.1 ± 45.1	138.8 ± 47.5	0.18
LAVI (ml/m2)	86.9 ± 19.7	81.1 ± 14.0	0.42
Conduction velocity (m/s) successful ablation site	0.75 ± 0.19	0.18 ± 0.05	0.04
Conduction velocity (m/s) endocardial successful and endocardial fail	0.75 ± 0.19	0.70 ± 0.16	0.48
Mitral isthmus mean bipolar voltage (mV)	1.33 ± 0.19	1.2 ± 0.26	0.32

BMI, body mass index; AF, atrial fibrillation; LA, left atrium; LV, left ventricle; LVEF, left ventricle ejection fraction; MV, mitral valve; CS, coronary sinus; TCL, tachycardia cycle length; AFL, atrial flutter; PV, pulmonary vein; VoM, vein of Marshall; SVC, superior vena cava; LAVI, left atrium volume index. The bold values are intended the significant findings.

There was no difference between the two groups according to the results of echocardiography parameters. Group 1 patients had a trend toward higher mitral A velocity than that in group 2, but *P*-value was not significant (61.8 24.8 vs. 47.6 15.7 cm/s, *P* = 0.075, respectively).

### Procedure characteristics

About 60% of patients had induced perimitral AFL after complete circumferential four PVI and substrate modification for persistent or long-standing persistent AF. Tachycardia cycle length was not significantly different between the two groups (Table 1). The CS activation sequence was distal to proximal as a clockwise perimitral AFL in 33 patients. Sixteen patients (88.9%) and seventeen patients (77.3%) received complete circumferential four PVI in groups 1 and 2, respectively.

**FIGURE 2:**
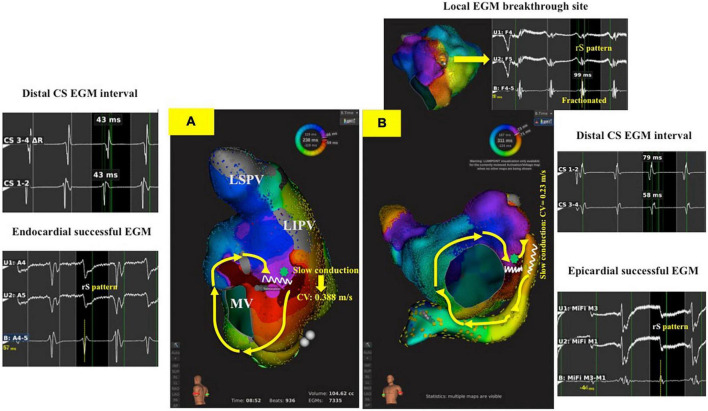
3-D activation map during perimitral AFL in endocardial and epicardial successful ablation groups. **(A)**: A patient with CW perimitral AFL and the AFL was successfully termination during posterolateral mitral line ablation with bidirectional block of mitral line (Group 1 patient). The interval of distal CS EGM was 43 ms, and the unipolar signal in endocardial successful site was rS pattern. The slowest conduction at endocardial success site (green star) in posterolateral mitral line was 0.388 m/s. **(B)**: A patient with CW perimitral AFL and the AFL was successfully termination during inside CS ablation with bidirectional block of mitral line following unsuccessful posterolateral mitral line ablation (Group 2 patient). The interval of CS 1-2 EGM was 79 ms, and the unipolar signal in epicardial successful site was rS pattern. The slowest conduction at epicardial success site (red arrow) in posterolateral mitral line was 0.23 m/s. The green star was focal breakthrough at posterolateral mitral line opposing to epicardial successful site showing fractionated EGM in bipolar and rS pattern in unipolar signal. AFL, atrial flutter; CS, coronary sinus; CW, counterclockwise; EGM, electrogram.

**FIGURE 3:**
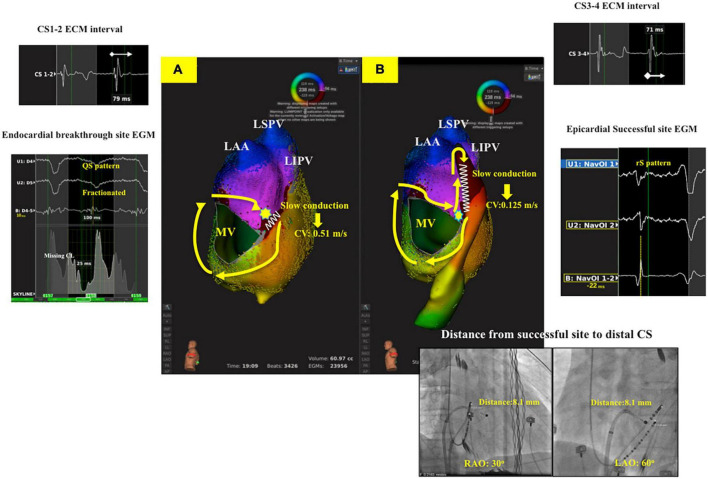
Ultra-high-density mapping during perimitral AFL in a patient with epicardial successful ablation (Group 2 patient). **(A)**: Activation map of LA showed clockwise perimitral AFL. Missing cycle length with color jump to epicardial site with focal breakthrough (Yellow star) at posterolateral mitral line with slow conduction (CV: 0.51 m/s). **(B)**: Activation map of LA and CS showed complete whole circuit of perimitral atrial flutter to epicardial region. Slow conduction was noted at distal CS (CV: 0.125 m/s) with the local unipolar signal at epicardial successful site revealing rS pattern, and the distance between ablation catheter to distal CS1-2 was 8.1 mm. The local EGM interval of distal CS was fractionated (CS1-2: 79 ms, CS3-4: 71 ms). LA, left atrium; CV, conduction velocity; AFL, atrial flutter; CS, coronary sinus; CW, counterclockwise; EGM, electrogram.

There was no significant difference between the two groups in LA volume, LAVI (Left atrium volume index). In group 2, the conduction velocity in epicardial successful site (intra-distal CS) was lower than that in the endocardial which was opposite to the epicardial successful site (0.690.14 m/s vs. 0.180.05 m/s; *P* = 0.01, respectively). The conduction velocity was slower at epicardial successful site (intra-distal CS) in group 2 compared to that at endocardial successful region in group 1 (0.18 0.05 m/s vs. 0.75 0.19 m/s; *P* = 0.04, respectively) (Figure 2). However, there was no significant difference in conduction velocity at endocardial ablation site of posterolateral mitral isthmus between groups 1 and 2 (Table 1). In group 2, seventeen patients had fractionated signal EGM (mean fractionated signal: 80.6 11.1 ms) in bipolar signal, and twenty patients had rS pattern and two patients had QS pattern in unipolar signal at focal breakthrough site opposing to successful epicardial site adjacent to distal CS (Table 2). Epicardial breakthrough was confirmed by missing cycle length or color jump to epicardial (mean missing CL: 28.5 4.8 ms and mean percentage: 11.72 4.81%) at activation map of LA during AFL. Local EGMs at epicardial successful sites showed rS pattern in 11 patients and QS pattern in three patients with median distance of 7.50 0.88 mm from ablation catheter to distal CS1-2 (Table 2). Figure 3 exhibits the ultra-high-density mapping during perimitral AFL in a patient with epicardial successful ablation and demonstrates the missing cycle length with color jump to epicardial site, and focal breakthrough at posterolateral mitral line. Slower conduction velocity and rS pattern of unipolar signal at epicardial successful site were found compatible with the results mentioned above.

**TABLE 2:** Electrophysiological characteristics at epicardial successful site.

Variable	Epicardial successful site (*n* = 22)
Tachycardia cycle length (ms)	237.7 ± 27.0
Focal breakthrough site at endocardial:	
1. Bipolar signal	Fractionated signal (*n* = 17)
2. Unipolar signal	rS pattern (*n* = 20), QS pattern (*n* = 2)
3. Mean fractionated signal (ms)	80.56 11.05
Missing cycle length (ms)	28.50 4.81
Percentage of missing cycle length (%)	11.72 1.74
Conduction velocity intra-distal CS successful site (m/s)	0.18 ± 0.05
Unipolar signal at intra-distal CS at successful site	rS pattern (*n* = 11), QS pattern (*n* = 3)
Distance from ablation catheter to distal CS (mm)	7.50 0.88

### Local coronary sinus interval electrogram

The local interval EGM at distal CS during perimitral AFL was longer in group 2 compared to group 1 at CS1-2 and CS3-4 (64.2 17.5 vs. 42.4 10.9 ms, 25.3 6.3 vs. 18.6 6.0 ms; *P* = 0.008, *P* = 0.001, respectively). Group 1 had a lower percentage of local EGM (local activation time/TCL) at the CS12, CS34, and CS12-34 compared to that in group 2 (18.6 6.0 vs. 25.3 6.3%, 19.2 5.4 vs. 23.7 6.8%, 22.2 6.3 vs. 29.0 7.6%, *P* = 0.001, *P* = 0.03, *P* = 0.004, respectively) (Table 1). There was no difference of local EGM interval and the percentage of local EGM at CS5-6, CS7-8, and CS9-10 between both groups (Table 1).

The multivariate analysis including all baseline characteristics, echocardiography parameters, procedure characteristics, and local EGM interval was used to identify the independent predictor for the need of epicardial ablation. A local EGM of CS1-2 interval was an independent predictor for epicardial success following endocardial failure in perimitral AFL (Hazard ratio: 1.33, 95% CI: 1.01 to 1.75, Table 3). A local EGM CS3-4 and CS12-34, and the percentage of local EGM CS1-2, CS3-4, and CS 12-34 were not the independent predictor of epicardial success (Table 3).

**TABLE 3:** Multivariate analysis.

Category	Univariate analysis				Multivariate analysis			
	HR	95% CI		*P*-value	HR	95% CI		*P*-value
		Lower	Upper			Lower	Upper	
Sex	5.00	0.87	28.96	0.72				
HTN	0.86	0.25	3.05	0.82				
Diabetes	0.35	0.06	2.18	0.26				
Dyslipidemia	1.26	0.19	8.52	0.81				
Smoking	0.78	0.16	3.67	0.75				
Thyroid diseases	0.26	0.04	1.54	0.14				
Coronary disease	0.35	0.06	2.18	0.26				
Renal failure	0.00	0.00	–	0.99				
AFL type	0.90	0.17	4.66	0.90				
Segmental PV	0.83	0.24	2.90	0.78				
Circumferential PV	0.47	0.12	1.76	0.26				
Anteromitral line	1.63	0.39	6.81	0.50				
Posterolateral ML	4.2	0.40	44.4	0.23				
PLML + AML	0.97	0.24	3.93	0.97				
Roof line	0.75	0.19	2.91	0.68				
CTI	0.76	0.18	3.62	0.71				
SVC isolation	0.36	0.03	4.30	0.42				
Intra-CS ablation	4.15	0.00	–	0.99				
VOM	2.07	0.00	–	0.99				
CL AFL	1.00	0.98	1.02	0.73				
CS1-2 interval	1.23	1.05	1.44	**0.01**	1.33	1.01	1.75	**0.04**
Percentage CS1-2	1.22	1.05	1.42	**0.01**	0.77	0.47	1.26	0.31
CS3-4 interval	1.08	1.00	1.17	**0.04**	–	–	–	–
Percentage CS3-4	1.12	1.00	1.26	**0.05**	–	–	–	–
CS12-34 interval	1.13	1.02	1.25	**0.01**	–	–	–	–
Percentage CS12-34	1.18	1.03	1.35	**0.01**	–	–	–	–
CS5-6 interval	1.00	0.95	1.07	0.88				
Percentage	0.98	0.87	1.10	0.74				
CS7-8 interval	1.00	0.96	1.05	0.75				
Percentage CS7-8	1.00	0.91	1.10	0.93				
CS 9-10 interval	1.00	0.97	1.04	0.60				
Percentage CS9-10	1.01	0.93	1.10	0.78				
Age	0.99	0.94	1.05	0.93				
LAD	1.01	0.93	1.10	0.76				
LVEF	0.95	0.83	1.08	0.44				
LVIDd (mm)	1.05	0.94	1.18	0.39				
LVIDs (mm)	1.04	0.93	1.17	0.48				
MV_E	0.99	0.96	1.01	0.27				
E/A	1.07	0.42	2.73	0.88				
MV_A	0.96	0.92	1.00	0.08	0.97	0.90	1.04	0.39
E’_Med	1.20	0.91	1.60	0.19				
E/E’ med	0.95	0.87	1.03	0.23				
LA volume	0.99	0.97	1.00	0.19				
LAVI (ml/m^2^)	0.98	0.93	1.02	0.41				
Compare CV endocardial success and endocardial failed	0.22	0.04	13.50	0.47				
Compare CV endocardial success and epicardial success	0.00	0.00	–	0.99				

The bold values are intended the significant findings.

**FIGURE 4:**
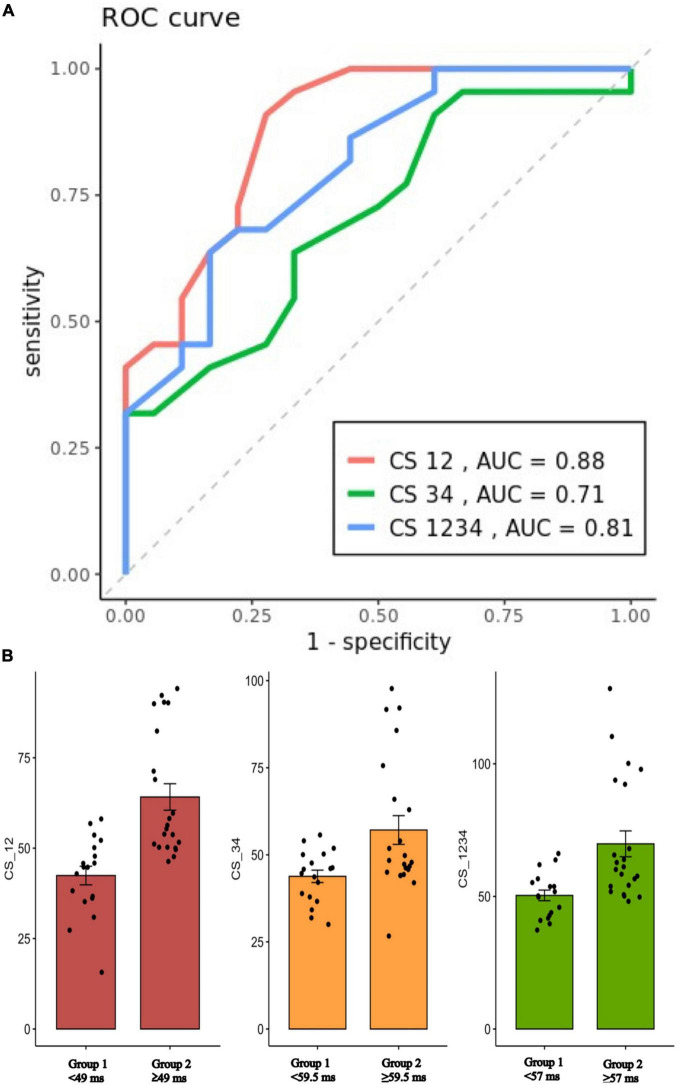
**(A)**: ROC curves for prediction of EEB on the CS12, CS34, and CS1234 interval (Cutoff value: 47, 59.5, and 57 ms, respectively). **(B)**: Scatterplot showing the mean value ± SD of the CS12, CS34, and CS1234 interval (ms) for patients with either group 1 (endocardial success) or group 2 (epicardial success). ROC, receiver operating characteristic; EEB, epicardial breakthrough; CS, coronary sinus; AUC, area under curve.

The optimal cutoff value of local EGM CS1-2 interval to discriminate the need of epicardial ablation for complete mitral line block was 49 ms with a sensitivity of 90.2%, a specificity of 72.2%, and AUC of 0.88 (Figure 4).

## Discussion

### Main finding

In our study cohort, the patients who need epicardial ablation (Group 2) had a longer EGM interval of distal CS than that in the patients who need epicardial ablation (Group 1). The conduction velocity at successful site was slower in group 2 compared to group 1 patients. In the multivariate analysis, distal EGM interval (CS1-2) was identified as independent predictor of the need of epicardial ablation.

### Epicardial breakthrough in perimitral atrial flutter

Previous studies had shown the epicardial connection in patients with perimitral AFL *via* CS and Marshall bundle. Catheter ablations for these cases were challenging, and endocardial ablation often failed to terminate the AFL and achieve mitral line block. Ablation intra-CS or VoM alcohol injection has been shown to be effective alternative to endocardial ablation alone (14, 18, 19). Discrimination of endocardial or epicardial connection during perimitral AFL had been an issue but lack of clinical predictor. de Groot et al. demonstrated the difference of unipolar electrogram morphology between epicardial breakthrough (rS pattern) and focal activation (QS pattern) (20). Pathik et al. suggested that (1) EEB sites were part of the circuit and critical to arrhythmia maintenance confirmed by entrainment, (2) and the demonstration of EEB during atrial macroreentry required the presence of an adjacent line of block or conduction slowing (21). Tung’s group demonstrated the features of epicardial breakthrough under ultra-high-density (UHD) mapping such as (1) discontinuous wave front of endocardial activation with a gap in activation leading to focal endocardial breakout distally and activation back toward the gap and (2) missing cycle length (>5% TCL) with detailed UHD mapping (4). The present study revealed the electrophysiological characteristics in patients with EEB during perimitral AFL, including lower conduction velocity and longer duration of local EGM in distal CS region.

### Mechanism of distal electrogram prediction epicardial breakthrough

Investigation of interatrial connection of Bachmann’s bundle in 1916 revealed the epicardial connection between interatrial connections at the anterior interatrial band named Bachmann’s bundle extends from the right of the superior vena cava transversally to the anterior wall of left atrial until the left atrial appendage (22). Lüdinghausen et al. investigated detail anatomic study documented the presence of a myocardial cover of the coronary sinus in 240 human hearts and the results displayed the connections with the posterior wall of the left atrium in 9% of cases; all hearts in the present histological study, which is based on serial sectioning, showed connections (23). The experimental study of CS-LA connections by Antz et al. demonstrated the centrifugal activation of the LA from discrete inputs originating from the CS musculature. Furthermore, the incisions isolating the ostium of the CS from the right atrium disconnected the CS and LA musculature, which initiated the electrophysiological role of these connections in maintaining LA activation (24). The other possibilities that the CS musculature forms another RA-LA connection are supported by surgical reports displaying that complete isolation of the LA in dogs and successful elimination of AF by the maze procedure required cryoablation of the muscular fibers of the CS (25–27). Antz et al. investigated the electrical activity of the CS musculature and its connection with the RA and LA may have multiple implications for the generation of atrial arrhythmia (24). The result showed that the CS musculature is electrically connected to the RA (*via* the CS ostium) and to the left atrium (distal LA-CS connection located 26 ± 7 mm from the ostium), forming an electrical RA-LA connection. Chauvin et al. proposed that the correlation between the left atrial myocardium and the CS muscle were divided by adipose tissue; this compartment tapered away from the ostium (0.86 ± 0.5 mm to 1.47 ± 1.2 mm) and was traversed by striated muscle fibers (28). No connection was between the coronary sinus musculature and left ventricular myocardium. The same study also revealed the anatomic and histological features of connection linking the right atrium to the left atrial myocardium tissue *via* a cuff of striated muscle around the coronary sinus in humans. The existence of this connection in all hearts examined indicates a consistent pathway for interatrial propagation (28). Kuo et al. showed that the elimination of distal CS to LA connections provides additional benefit to standard PV isolation and non-PV trigger ablation in reducing atrial arrhythmia recurrences in AF patients referred for catheter ablation (15). In agreement with the aforementioned studies, our study disclosed that the conduction velocity of intra-distal CS at the successful site was slower compared to other regions at LA myocardium.

In patients who need epicardial ablation, the epicardial successful ablation sites in the lateral mitral isthmus were close to distal CS1-2 with median distance of 7.50 ± 0.88 mm (Table 2). Activation mapping of left atrial endocardium during perimitral AFL demonstrated the missing cycle length with color jump to epicardial site of lateral mitral isthmus suggesting a transmural conduction. Our study showed that epicardial successful ablation site adjacent to the intra-distal CS had a slower conduction velocity. Therefore, longer endocardial-to-epicardial conduction time with slower velocity in the epicardial successful ablation site may be responsible for the longer EGM interval recorded in distal CS during perimitral AFL in patients who need epicardial ablation. Moreover, the conduction velocity at epicardial successful sites was lower than that in the corresponding endocardial area. Patients in epicardial successful group had a lower conduction velocity of intra-distal CS than those in endocardial successful group. The epicardial successful ablation sites (epicardial bridging site) in the lateral mitral isthmus were close to distal CS1-2, which was also near the vein of Marshall (VOM). Both intra-distal CS and VOM are the adjacent structure of epicardial bridging site. In group 2 patients, the electrical activation traveled from endocardium to epicardium and then conducted to endocardium which might cause a longer conduction time, slow velocity, and fractionated EGM. However, group 1 patients had purely endocardial conduction without epicardial bridging. As a result, the conduction velocity at the successful site was slower in group 2 compared to that in group 1.

To the best of our knowledge, the present study is the first study demonstrating that the local EGM interval of distal CS (cutoff value: 49 ms) can predict the possibility of epicardial breakthrough in perimitral atrial flutter.

### Study limitations

Our study was limited by a small sample size of the patients and single center. The retrospective nature of this study encompasses significant heterogeneity of the data collected, especially when different mapping systems (up to three) are used in this study population. The elaboration of prediction models with univariate and multivariate analyses based on retrospective data in a limited number of patients might restrict the widespread applicability and reproducibility of these results. Most of the patients had multiple prior ablation procedures, and 55% with epicardial breakthrough have undergone more than two prior ablation procedures which may affect the local EGMs and conduction velocity. LA substrate including the low voltage areas and complex electrogram plays an important role in maintaining the macroreentry activation. The mitral isthmus often serves as an anatomic isthmus instead of an electrophysiological isthmus with normal substrate. Therefore, linear ablation based on LA substrate should be considered. Although the outcome of short-term follow-up is good, the long-term outcome could be a problem because the substrate was still there. Furthermore, it is difficult to identify a discrete and clear successful ablation site for conduction velocity measurement in all cases, especially when block to the mitral isthmus line was achieved by means of VOM ethanol infusion.

## Conclusion

Longer EGM interval in distal CS during perimitral AFL was observed in perimitral AFL patients with epicardial breakthrough following endocardial-failed ablation. Slow conduction and longer EGM interval of distal CS during perimitral AFL may be associated with the need of epicardial ablation. A prospective study is warranted to prove this finding.

## Data availability statement

The original contributions presented in this study are included in the article/supplementary material, further inquiries can be directed to the corresponding author.

## Ethics statement

The studies involving human participants were reviewed and approved by Institutional Review Board at Taipei Veterans General Hospital, Taipei, Taiwan. Written informed consent for participation was not required for this study in accordance with the national legislation and the institutional requirements.

## Author contributions

All authors listed have made a substantial, direct, and intellectual contribution to the work, and approved it for publication.
